# Supplementary material for: Spatial Heterogeneity and Methodological Insights in Fish Community Assessment: A Case Study in Hulun Lake
Source: Biology (Basel). 2025 Nov 26;14(12):1678. doi: 10.3390/biology14121678 (PMC12730304; doi:10.3390/biology14121678)
Supplement: Supplementary file 1 [file biology-14-01678-s001.zip › supporting_informations-Figures.pdf]

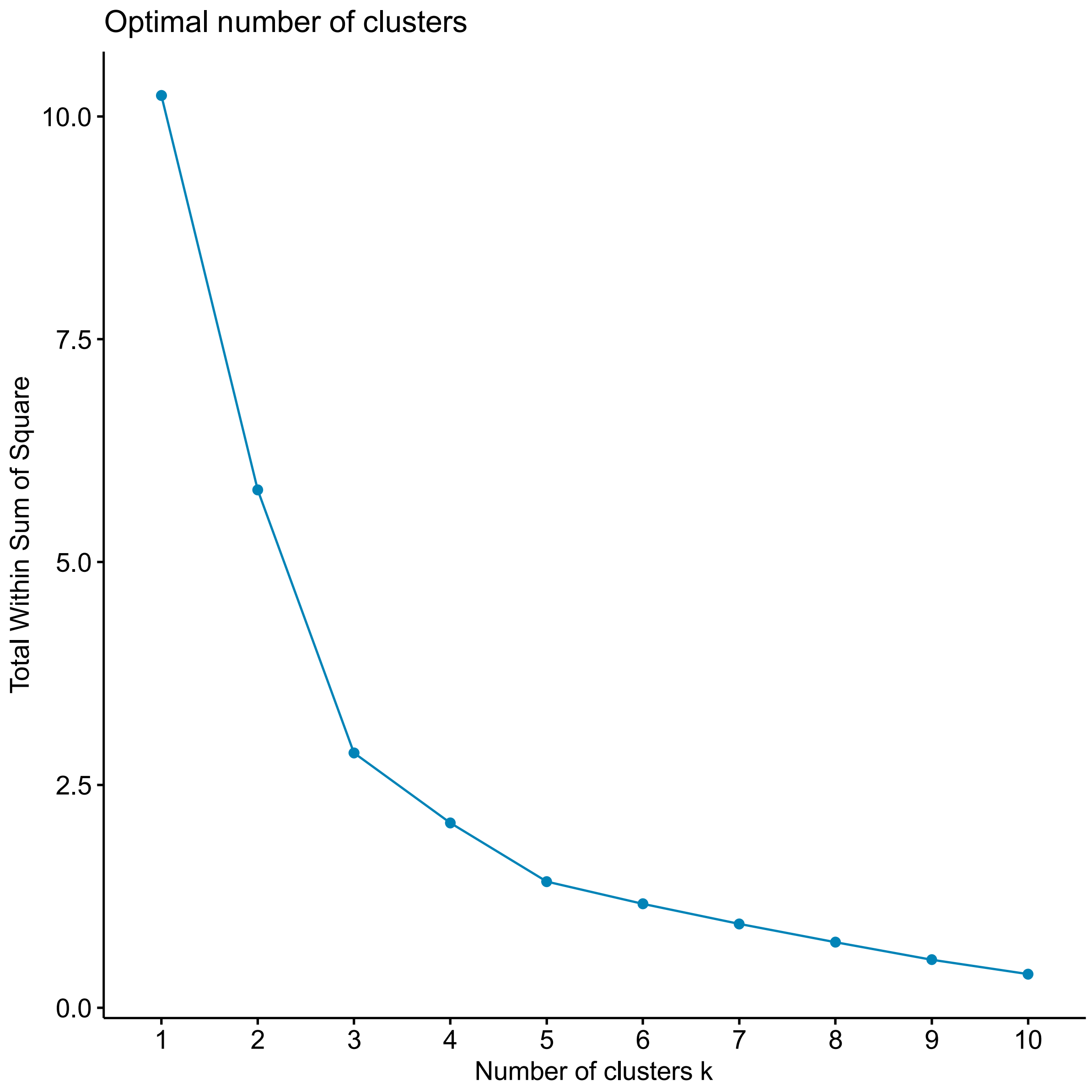

Figure S1. Elbow plot for determining the optimal number of spatial clusters for fish community in Hulun Lake. The 'elbow' at k = 4 suggests the most appropriate number of clusters, balancing within-cluster variance and model simplicity.

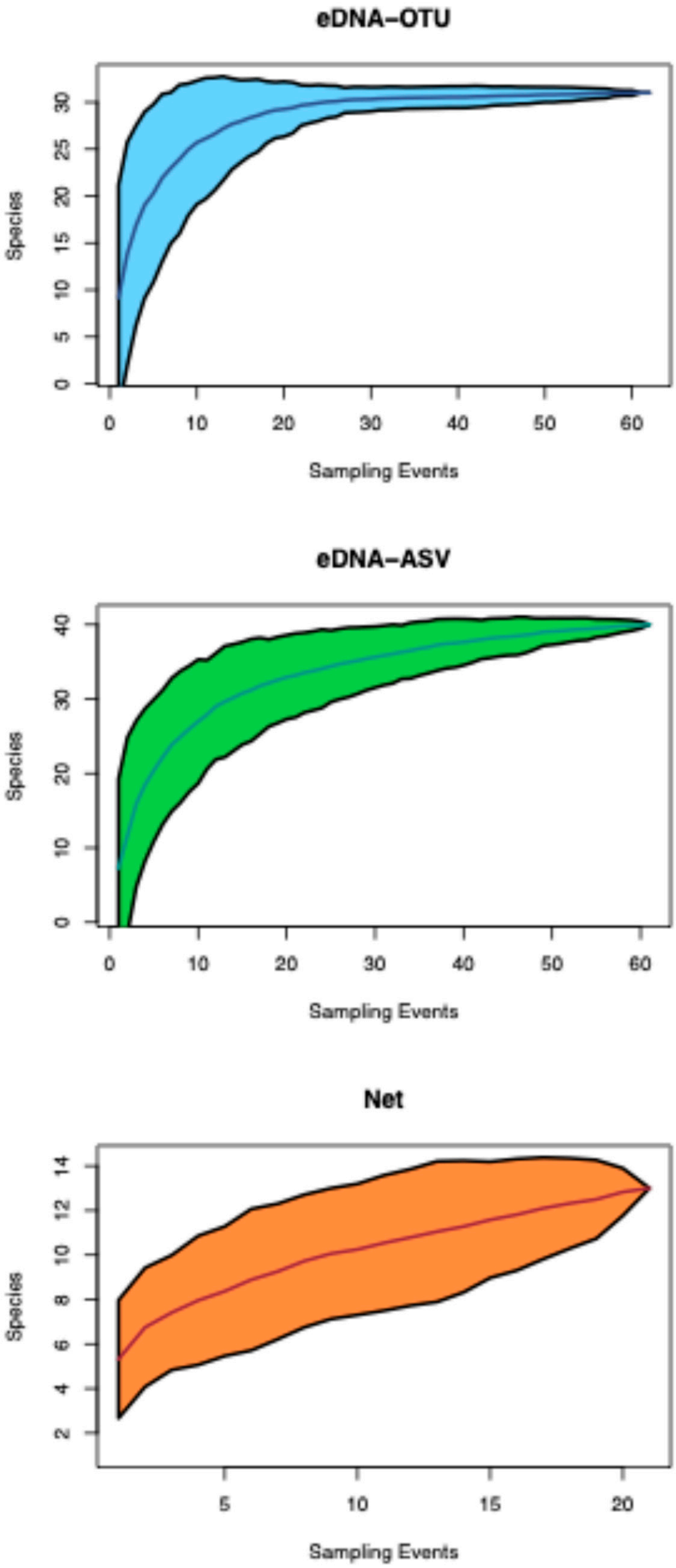

Figure S2. Accumulation curve, calculated based on OTU and ASV based eDNA datasets and capture-based survey data, using “speccum” function with “random” method.

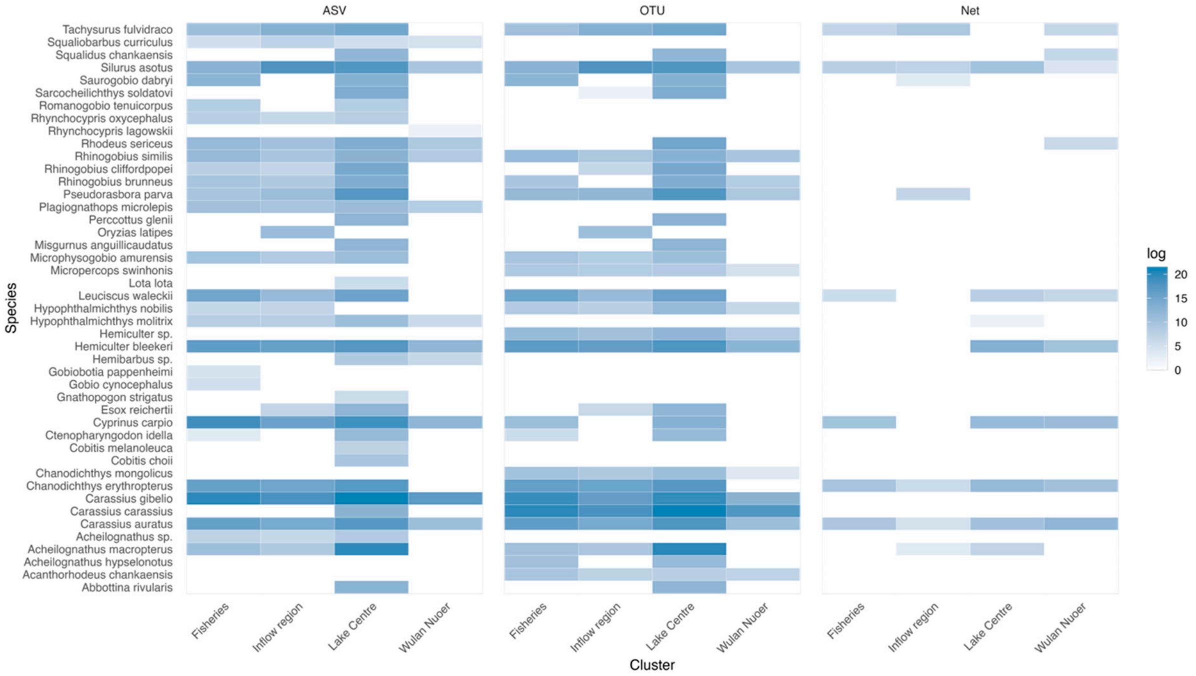

Figure S3. Heatmap of species abundance (log2 transformed) in each cluster.

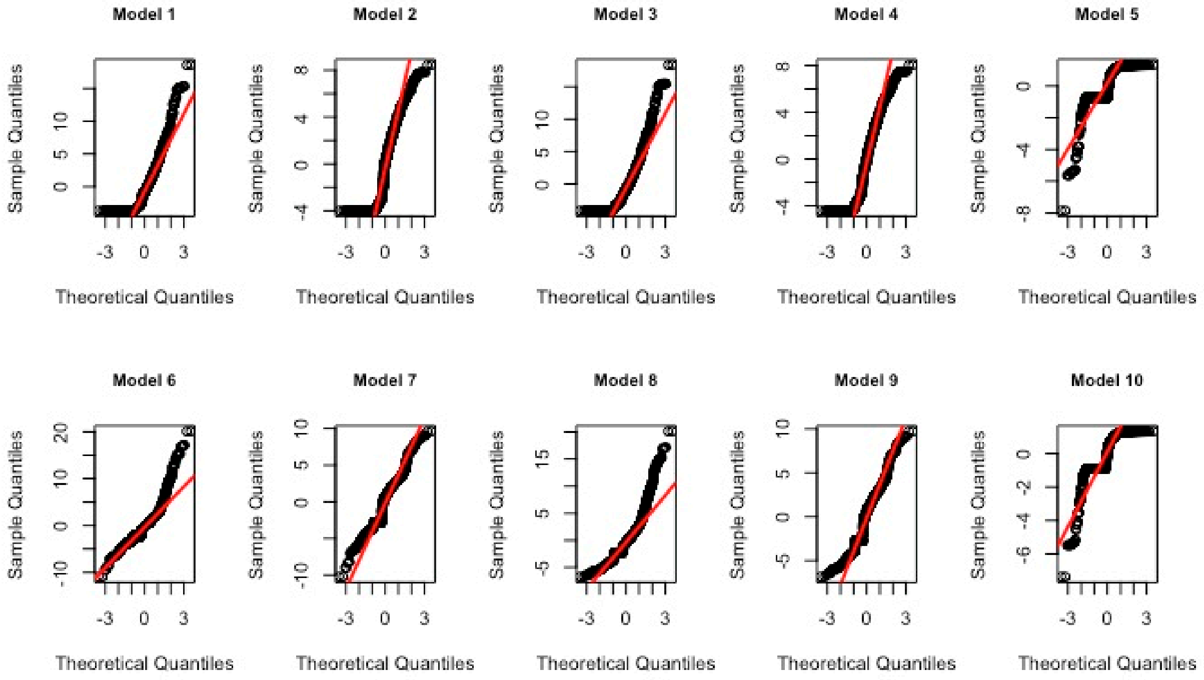

Figure S4. Q–Q plots of residuals for linear regression models. Each panel shows the quantiles of model re-siduals plotted against theoretical normal quantiles. The red reference line represents a perfect normal distribution.
